# Accelerated Hydrolytic Degradation of PLA/Magnesium Composite Films: Material Properties and Stem Cell Interaction

**DOI:** 10.3390/polym17152052

**Published:** 2025-07-27

**Authors:** Valentina Fabi, Maria Luisa Valicenti, Franco Dominici, Francesco Morena, Luigi Torre, Sabata Martino, Ilaria Armentano

**Affiliations:** 1 Department of Economics, Engineering, Society and Business Organization (DEIM), University of Tuscia, Largo dell’Università snc, 01100 Viterbo, Italy; valentina.fabi1@studenti.unitus.it; 2 Department of Chemistry, Biology and Biotechnologies, University of Perugia, 06123 Perugia, Italy; marialuisa.valicenti@dottorandi.unipg.it (M.L.V.); francesco.morena@unipg.it (F.M.); 3 Department of Civil and Environmental Engineering, University of Perugia, UdR INSTM, Strada di Pentima 4, 05100 Terni, Italy; franco.dominici@unipg.it (F.D.); luigi.torre@unipg.it (L.T.)

**Keywords:** magnesium, degradation, stem cells

## Abstract

The accelerated hydrolytic degradation of poly(L-lactide) (PLA)/magnesium (Mg) composite films was investigated to elucidate the influence of surface modification of Mg particles on the degradation behavior and characteristics of PLA composites. Accelerated degradation studies were conducted at 60 °C in a pH 7.4 phosphate-buffered solution over 7 weeks, with degradation monitored using several techniques: mass loss, water absorption, thermal analysis, and Raman spectroscopy. The results indicated that all composite films experienced more than 90% mass loss at the end of experiment; however, PLA/5MgTT and PLA/5MgPEI exhibited the highest resistance to degradation, likely due to the protective effect of the surface modification induced by thermal treatment and polyethylenimine (PEI). Notably, these characteristics did not compromise the biocompatibility or osteogenic potential of the films, which remained comparable to the control samples when tested on human bone marrow multipotent mesenchymal/stromal cells.

## 1. Introduction

Poly-lactic acid (PLA) composites, obtained by combining the PLA polymer matrix with micro- and/or nano-fillers, result in biomaterials with properties distinct from those of the individual components. These materials have received much attention due to their versatile properties, which allow many applications in a huge number of fields. In particular, the addition of magnesium particles has also emerged in the last years as a very interesting approach, especially in biomedical fields, for their potential use in the repair of bone tissue, due to the combination of the properties of PLA polymers with the osseointegration and osteoconductive potentials of magnesium (Mg) [1,2].

Magnesium (Mg) is one of the most abundant intracellular divalent cations and an essential metal in the human body. Mg plays a key role in bone health [3], as it stimulates osteoblast proliferation [4] and plays a role as an enzyme cofactor in bone matrix synthesis [5]. Several studies have demonstrated a synergistic interaction between PLA and Mg [1]. Specifically, while PLA slows the high rate of Mg degradation [6], Mg helps to neutralize the acidic environment formed by PLA degradation by-products, enhancing cell compatibility through the release of Mg ions [2,7]. On the other hand, the microstructure of the PLA interface can significantly affect the properties of Mg-based composite films [8]. Therefore, the effectiveness of PLA/Mg composites for biological applications depends either of the characteristics of the polymer film or on the type of Mg used (e.g., Mg ions, Mg particles, Mg alloys) [2,7,9] and, most importantly, on the interface properties between polymer and Mg particles. In this context, some studies have shown that modifying Mg particles through surface adsorption treatments positively influences the biodegradation of PLA/Mg films [2], although certain mechanisms, such as the impact of surface modifications on the degradation kinetics of biodegradable polymers, remain not fully understood. Mg fillers influence pH reduction and alter the hydrolytic degradation of the polymers.

The hydrolytic degradation of PLA is an important factor to consider for a variety of biomedical applications, such as tissue engineering and drug delivery. It occurs by the uptake of water by the hydrolysis of the ester bonds. Surface erosion and bulk degradation are the two possible mechanisms identified [10,11]. Previous studies demonstrated that the hydrolytic degradation of bulk amorphous poly(D, L-lactic acid) samples should proceed heterogeneously; it is faster at the inner parts respect at the surface and its autocatalysis may affect the interior part more [12]. Different factors affect the hydrolysis kinetics of PLA, such as the water diffusion process and kinetics, the temperature, and the pH. Furthermore, the crystalline phase presence, the glass transition of the amorphous phase, and the surface hydrophobicity have important effects on the degradation rate. Different methods have been used to track the progress of the degradation process such as thermal analysis, spectroscopies, molecular weight evaluations, etc. [13,14].

In this study, we conducted an in-depth monitoring of the accelerated degradation behavior of both pristine poly(lactic acid) (PLA) and PLA/Mg composites over time. To reduce experimental time and associated costs, accelerated degradation tests were carried out at 60 °C, with all tested material in the rubbery state, in accordance with previous reports [11,15]. In this way, the degradation experiments do not replicate the state of the materials during the biomedical applications. To obtain appropriate and significant knowledge on the long-term behavior of polylactide polymer devices, artificially accelerated degradation tests are selected as the favored method in both research and industrial practice, even if this procedure does not accurately replicate real-time degradation [11,16]. Furthermore, previous results suggest the feasibility of performing accelerated degradation assays in order to extrapolate aliphatic polyester degradation at operation conditions [17,18].

Raman spectroscopy, a non-destructive, highly informative technique for analyzing chemical composition, molecular configuration, and structural conformation, was employed to monitor the evolution of the materials during degradation.

PLA-based composites were prepared by incorporating 5 wt.% Mg, and the influence of Mg, with particular focus on different surface modifications, was investigated in terms of its effect on the degradation kinetics of PLA. The impact of elevated immersion temperatures on the degradation behavior of both neat PLA and PLA/Mg composites was quantitatively assessed. In addition, the biological response was evaluated by culturing human bone marrow-derived multipotent mesenchymal/stromal cells (hBM-MSCs) on the PLA/Mg-based films.

These findings highlight the potential of tailored PLA/Mg composites for use in bioresorbable implants, where controlled degradation rates and favorable cellular interactions are essential for clinical success.

## 2. Materials and Methods

### 2.1. Materials

Commercial poly(lactic acid) grade 2003D, having a specific gravity of 1.24 g⋅cm^−3^, molecular weight (Mn) 1.42 × 10^4^ g·mol^−1^, and melting flow rate (MFR, 210 °C, 2.16 kg loading) of 6 g/10 min, was purchased from Nature Works^®^, Co. LLC, Minnetonka, MN, USA. Magnesium (Mg) particles were supplied by Nitroparis^®^, Paris, Ile-de-France, France. They had spherical morphology with a diameter ranging from 10 μm to 50 μm, with the presence of satellite particles [19].

Polyethylenimine (PEI), Mv 25,000, was supplied by Sigma Aldrich^®^, St. Louis, MO, USA.

### 2.2. Composite Development

PLA-based composite films had been developed by using Mg particles at 5 wt.%, selected according to previous works [1,20]. Mg particles were surface-modified by using two different treatments: thermal treatment (MgTT) at 350 °C in air for 1 h and PEI adsorption (MgPEI). PEI, a cationic dispersant, was selected, taking into account the negative charge of Mg particles in aqueous media, in order to permit the formation of a strong PLA–PEI interface during composite development [21].

Composite films consisting of 5 wt.% of different treatments of Mg particles were processed in a twin corotating screw microextruder (Dsm Xplore 5&15 CC Micro Compounder, Sittard-Geleen, The Netherlands) and designated as PLA, PLA/5Mg, PLA/5MgTT, and PLA/5MgPEI. The temperature profile was set up as 180–190–200 °C with a screw speed of 120 rpm and a mixing time of 3 min, according to the thermal properties of the selected PLA polymer.

### 2.3. Hydrolytic Degradation

Accelerated hydrolytic degradation was carried out in 0.1 M phosphate-buffered solutions (PBS) at pH 7.4 ± 0.2 [14] for 50 days [22]. Specimens with dimensions of 30 mm × 10 mm and a thickness of 50 μm were placed into small flasks filled with 5 mL of 0.1 M PBS solutions [14]. The flasks were allowed to stand in a thermostatic oven (ISCO thermostatic laboratory heater) set at 60 °C for 49 days. This method was selected since it permits one to accelerate the hydrolysis of PLA-based materials, even if it does not simulate the real state of the materials during the biomedical applications. At each degradation time, three specimens were withdrawn and washed with distilled water. The immersion fluid was changed every day and, prior to the change, the pH was measured.

### 2.4. Material Characterization

#### 2.4.1. Visual Observation

The PLA-based composite film decomposition was firstly analyzed via visual inspection by using a photographic camera (Nikon D 5300, Nikon Corporation, Tokyo, Japan) without filter and in automatic exposure mode. The pictures were then visually analyzed at each time, to compare the effects of degradation at macroscopic level. In particular, the appearance of pores, streaks, or other modifications on the PLA stripes were observed and described.

#### 2.4.2. Weight Loss and Water Adsorption

Wet samples were weighed immediately after removing them from the solution and their surface was dried with a paper towel; dry samples were measured after keeping them 5 h in the oven at 60 °C. Two types of measurements were carried out: mass variation and water intake. A precision balance (Gibertini E42S-B, Gibertini Elettronica S.r.l., Novate Milanese, Italy) was employed to weigh all samples within an error of 0.0001 g. Experiments were conducted in triplicate. The loss of mass due to degradation was recorded at each timepoint [22] and the mass loss percentage (*WL*%) was calculated by Equation (1):

(1) $$WL\%=\left(\frac{w_{o}-w_{f}}{w_{0}}\right)\times 100$$

where *w_o_* and *w_f_* are, respectively, the initial and the final mass (g) of the PLA-based samples. The average of each calculated percentage for both samples of each type of PLA at each time was then calculated.

The percentage of water adsorption (*WA*%) was calculated by comparing the weight of the wet sample with the weight of the same sample after oven-drying. The water intake measurements were obtained through the difference between the mass of the wet specimen *m_b_* and the mass of the same specimen after letting it dry *m_s_*, as reported in Equation (2):

(2) $$WA\%=\left(\frac{m_{b}-m_{s}}{m_{b}}\right)\times 100$$

where *m_b_* is the wet mass, whereas *m_s_* is the dry mass (g), i.e., after the sample was oven-dried.

#### 2.4.3. Thermogravimetric Analysis

The thermal degradation of degraded PLA-based samples was evaluated by thermogravimetric analysis (TGA, Seiko Exstar 6300, Tokyo, Japan) by thermal dynamic tests from 30 °C to 600 °C at 10 °C min^−1^, under nitrogen flow (200 mL min^−1^). Mass loss (TG) and derivative mass loss (DTG) curves for each tested material were evaluated.

#### 2.4.4. Raman Spectroscopy

A Horiba Jobin Yvon spectrometer (XPlora^TM^, Palaiseau, France) was used for the Raman spectroscopy of the PLA-based samples. A 532 nm laser (25 mW power) with an Olympus^®^ BX41 microscope, Olympus Corporation, Tokyo, Japan, equipped with 50× objective Olympus, was used for spectra acquisition. All spectra were detected by means of a CCD detector, 2 cm^−1^ hardware resolution. The laser at wavelength 532 nm was used at 50% maximum power. The instrument was calibrated with the Rayleigh line at 0 cm^−1^ and standard Si(100) reference band at 520.7 cm^−1^. The acquisition parameters were set at 5 s of excitation time (ET) and 30 accumulations in the Acquisition tab. Spectral data were acquired and elaborated by LabSpec6^®^ dedicated software. Pristine and degraded samples were analyzed.

### 2.5. Culture of Multipotent Human Mesenchymal/Stromal Cells on PLA and Composite Films

#### 2.5.1. Multipotent Human Mesenchymal/Stromal Cells

The interaction of PLA film and PLA/5Mg, PLA/5MgTT, and PLA/5MgPEI composite films were assessed by their incubation with human bone marrow multipotent mesenchymal/stromal cells (hBM-MSCs).

hBM-MSCs, already available in our laboratories [23,24,25], were cultured in RPMI-1640 (Euroclone, Pero (MI), Italy), the growth culture medium complemented with 10% heat-inactivated FBS (Euroclone, Pero (MI), Italy), 1% penicillin/streptomycin, and 1% L-glutamine (Euroclone, Pero (MI), Italy), by seeding in a tissue culture polystyrene flask (TCP) and then incubated at 37 °C, under 5% CO_2_. hBM-MSCs grew as adherent fibroblast-like cells and the culture medium was changed every three days [19].

#### 2.5.2. Sterilization of Films and Seeding of Stem Cell

The films (PLA and composites PLA/5Mg, PLA/5MgTT, and PLA/5MgPEI) were cut into 1 cm^2^ quadrangles and sterilized by immersing them in ethanol at 70% for ten seconds. They were then rinsed three times with PBS to remove any remaining ethanol and placed in a 24-well plate. The cell suspension containing 1.5 × 10^3^ cells was prepared, then was carefully applied dropwise onto the sterilized films, and 500 μL of growth medium was progressively added to each well and cultured as described above. As an internal control, stem cells were also planted on glass coverslips (GCs) and TCP, which served as reference materials. PLA-derived stem cell cultures were assessed at different time points for cell viability, morphology, and osteogenic differentiation [24,26,27].

#### 2.5.3. MTT Assay

The viability of hBM-MSCs on pure PLA and on the composite films PLA/5Mg, PLA/5MgTT, and PLA/5MgPEI was assessed at different time points (3, 7, 14, 21) with the MTT assay (3-(4,5-dimethylthiazol-2-yl)-2,5-diphenyltetrazolium bromide) (Sigma-Aldrich, St. Louis, MO, USA) following the producer’s recommendations. Stem cells on TCP were used as a control. Additionally, the possible interference of each film square on the MTT assay was evaluated without the presence of hBM-MSCs. The absorbance of samples at 589 nm with a reference wavelength of 650 nm was measured by the ELISA reader, GDV-DV990BV6, Rome, Italy [19,24].

#### 2.5.4. Osteogenic Differentiation

To evaluate the osteogenic potential of Mg on composite polymer films, hBM-MSCs were plated on sterilized PLA, as well as PLA/5Mg, PLA/5MgTT, and PLA/5MgPEI composite films in the growth culture medium. As a positive control (CTR+), the osteogenic differentiation was induced using commercial hMSC differentiation basal medium supplemented with osteogenic components SingleQuotsTM (Lonza Walkersville, Inc., Walkersville, MD, USA), which included dexamethasone, L-glutamine, ascorbate, penicillin/streptomycin, mesenchymal cell growth supplement, and β-glycerophosphate. As a negative control (CTR−), untreated cells were cultured in growth medium. The overall cultures were sustained for 21 days in the above culture condition, with the medium being replaced every 3 days.

#### 2.5.5. Osteogenic Differentiation Assays

##### Alizarin Red Staining

According to our previous works [23,28], on day 21 of culture, cells on PLA, PLA-composite films, and TCP (CTR+ and CTR−) were washed with PBS, fixed in 4% paraformaldehyde for 20 min, and, with distilled water, incubated with 500 μL of Alizarin Red solution (Lonza Walkersville Inc., Walkersville, MD, USA) for 30 min at room temperature (RT). Cells were then further washed with distilled water, and the images captured using a Canon digital camera (PowerShot G10, Canon, Tokyo, Japan) and brightfield microscopy (Eclipse-TE2000-S, Nikon Tokyo, Japan).

##### Alkaline Phosphatase Staining

Alkaline phosphatase (ALP) staining was performed according to previous work [19,28]. Cells on PLA, PLA-composite films, and TCP (CTR+ and CTR−) were washed twice with PBS, fixed in 4% paraformaldehyde for 20 min, rinsed with distilled water, then incubated with 500 μL of FAST BCIP/NBT substrate solution (Sigma-Aldrich, St. Louis, MO, USA) for 2 h at room temperature. After the incubation, all samples were washed with distilled water and images were acquired using a Canon digital camera (PowerShot G10, Canon, Tokyo, Japan) and a brightfield microscope (Eclipse-TE2000-S, Nikon).

#### 2.5.6. Immunofluorescences

The immunostainings were performed as described [19,24,27,28]. In brief, cells on PLA, PLA-composite films, and glass coverslips (CTR+ and CTR−) were washed with PBS, fixed in 4% paraformaldehyde for 20 min, rinsed with PBS, and permeabilized in a solution of PBS with 3% FBS and 0.5% Triton X-100 for 30 min at RT. Cells were then incubated in a blocking solution (PBS with 3% FBS and 0.05% Triton X-100) for 1 h at RT. Stem cell morphology was evaluated by incubating cell samples with Alexa-Fluor-488 phalloidin (Invitrogen, Grand Island, NY, USA) for 20 min for F-actin detection.

To assess osteogenic differentiation cells cultured on PLA, PLA-composite films, and glass coverslips (CTR+ and CTR−), samples were incubated O/Nt at 4 °C with primary antibody anti-type I Collagen (COL.1) (Chemicon International, Temecula, CA, USA). Finally, after PBS washing, all samples were mounted, and nuclei were counterstained with Vectashield^®^ containing DAPI (4,6-diamidino-2-phenylindole; Vector Laboratories, Burlingame, CA, USA). Immunofluorescence was evaluated using an Eclipse-TE2000-S fluorescence microscope (Nikon, Tokyo, Japan) equipped with an F-View II FireWire camera (Soft Imaging System, Olympus, Germany) [19].

### 2.6. Statistical Analysis

The data analysis is presented as mean ± standard deviation (SD). A post hoc comparison was conducted using the one-way ANOVA and Dunnett’s Multiple Comparison Test (GraphPad v.10, San Diego, CA, USA). A *p*-value of less than 0.05 was regarded as statistically significant. The significance of the differences is indicated as follows: * *p* < 0.05; ** *p* < 0.01; *** *p* < 0.001; **** *p* < 0.0001.

## 3. Results

### 3.1. PLA-Based Composites

PLA composite films with 5 wt.% of Mg particles modified by using two surface treatments were successfully developed by an extrusion process with the selected temperature and persistence time. Obtained films are homogenous, 35 mm wide, and with a thickness ranging between 40 and 100 μm. The Mg thermal treatment permits one to change the surface layer of the Mg particles, which can modify the interface and the final composite film properties. The MgTT particles increase surface roughness, with the presence of small cracks and pores, by maintaining the spherical shape, while the PEI creates an appropriate interface between PLA and Mg particles, improving the dispersion of the metallic phase inside the polymer matrix [19].

The PLA films are characterized of a glass transition of around 56 °C, a T_cc_ at 118.5 °C, and finally a T_m_ at 148.3 °C, with almost a completely amorphous behavior. PLA composites exhibited similar thermal characteristics [19]. The introduction of Mg particles modulated both the bulk and surface properties of the PLA polymer-based films. In particular, thermal stability decreases with the presence of magnesium in the PLA polymer and reduces the polymer chain mobility as a result of the interface properties on the Mg particle surface, as underlined by DSC behavior [19]. Moreover, the processed PLA composites maintained the PLA Young’s modulus and modified the surface properties, increasing the wettability properties of the PLA films.

### 3.2. Hydrolytic Degradation

#### 3.2.1. Visual Observation

**Figure 1:**
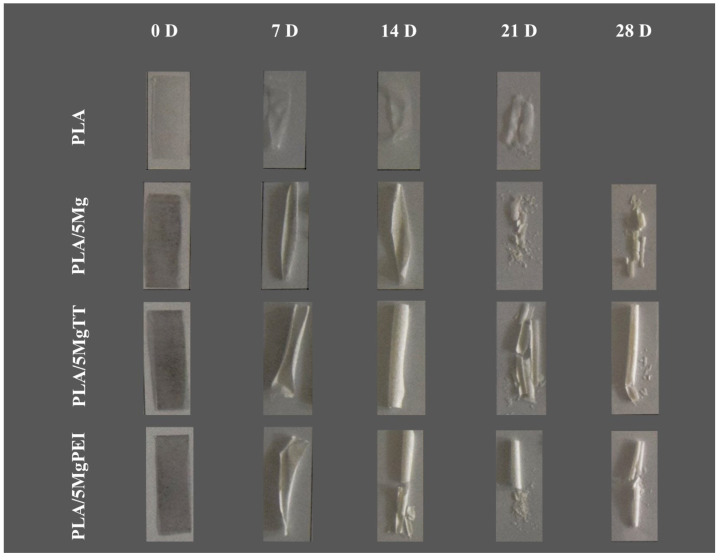
PLA, PLA/5Mg, PLA/5MgTT, and PLA/5MgPEI films before and after the degradation in PBS at 60 °C at 7, 14, 21, and 28 immersion days.

Figure 1 shows the images of the specimens before and after hydrolytic degradation in PBS at 60 °C for 7, 14, 21, and 28 immersion days. During the hydrolytic degradation, all developed materials are in the rubbery state. The PLA-based film presents an increase in the relative opacity against degradation time at relatively short times, as well as an extreme brittleness for all the materials after the first week. Mg-treated PLAs showed higher resistance properties, like PLA/5MgTT and particularly PLA/5MgPEI. These materials showed a minor degradation-induced brittleness at the end of the degradation process; meanwhile, pristine PLA rapidly degraded. PLA/5Mg showed an intermediate behavior. It is known that the presence of Mg particles has important consequences on PLA polymers, in particular the optical properties such as transparency and color, surface properties such as roughness, and bulk properties such as mechanical and thermal [19]. In our study, the discoloration of the magnesium-containing samples was clearly observed. Initially darker, these samples progressively assumed a lighter color during the accelerated degradation process, as a consequence of magnesium degradation. Similarly, pristine PLA, which appeared transparent at the beginning of the test, rapidly changed color and completely disintegrated after only 28 days. The whitening of the surface over time is attributed to significant surface degradation induced by hydrolysis, resulting from moisture adsorption and the formation of degradation byproducts, which cause changes in the material’s refractive index [29]. The observed opacity, resulting from degradation, may be attributed to various phenomena, such as light scattering caused by degradation products formed during the hydrolytic process, the formation of voids within the bulk of the PLA samples during degradation, or changes in the PLA crystallinity [30,31]. The hydrolytic degradation of polyester chains is observed to proceed at a higher rate in the amorphous regions of the polymer. In the case of PLA/5Mg, degradation primarily occurs at the surface of the sample; as the hydrolytic degradation time increases, the inner parts of the sample also begin to degrade [32].

#### 3.2.2. Mass Loss

**Figure 2:**
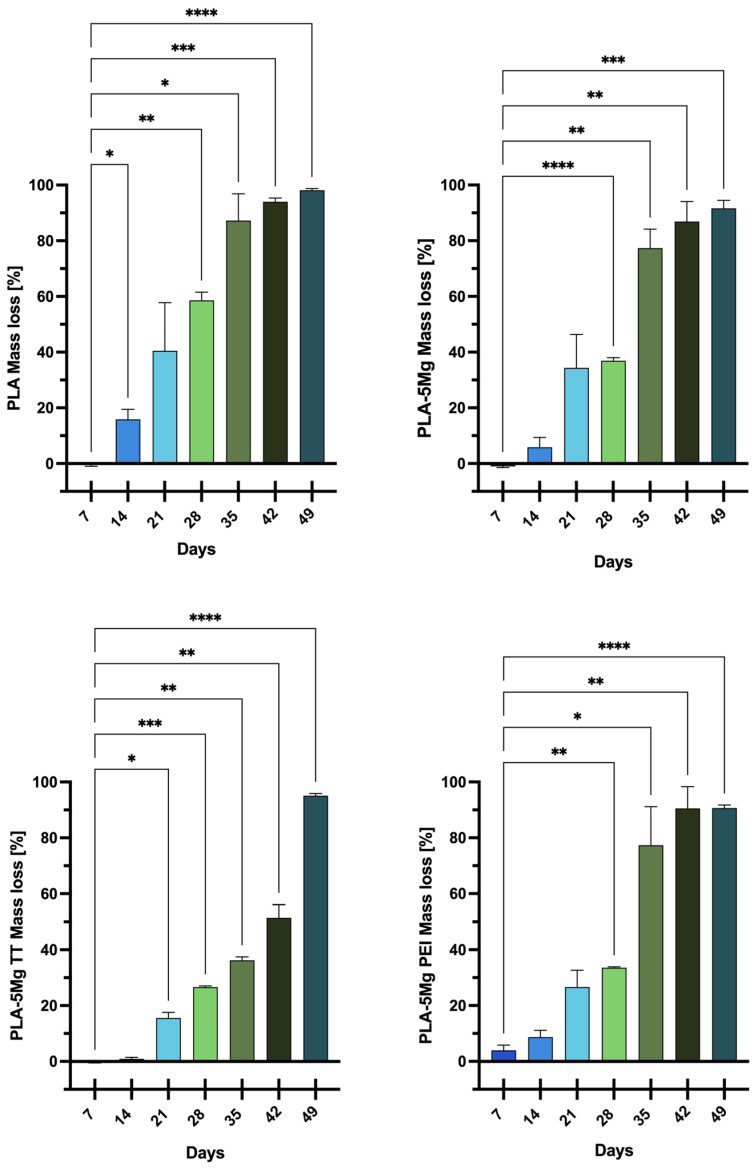
Mass loss for PLA, PLA/5Mg, PLA/5MgTT, and PLA/5MgPEI PLA films as functions of degradation time in PBS at 60 °C. Results are expressed as mean ± SD of three independent experiments. The significance of the differences is indicated as follows: * = *p* < 0.05; ** = *p* < 0.01; *** = *p* < 0.001; **** = *p* < 0.0001.

Figure 2 reports the weight loss of the samples, expressed as a mean percentage of the corresponding 0-days weight, as a function of the days for all the analyzed samples. Pristine PLA weight, after the first week, did not decrease significantly, but after just 14 days it showed a significant mass loss compared to PLA composites, corresponding to about 16 ± 4%. After 21 days, PLA samples reach 40% of the mass; Mg-based composites show similar values (34%), while important differences were obtained for Mg-treated composites, with 27% for PLA/5MgPEI and 16% for PLA/5MgTT. The mass loss decreased gradually during the degradation process, reaching a value of about 98 ± 1% at the end of the tests, although pristine PLA samples revealed the highest mass loss during all the phases of the degradation process. PLA/5Mg underwent a small change in mass between 7 days and 14 days. The weight loss then increased after 21 (34 ± 12%) and 28 days (37 ± 1%): the mass loss was more than one third of the initial one after 28 days. Mass loss continued to increase, reaching a very high value of 92 ± 3% after 49 days. PLA/5MgTT samples were stable until 14 days. The weight loss increased rapidly and constantly from 21 days (16 ± 2%) until 42 days and, at the end of the experiment, the mass loss was very high (95 ± 1%), comparable to that observed for PLA/5Mg. Finally, PLA/5MgPEI underwent a small change in mass between 7 and 14 days too. As observed for PLA/5MgTT, the weight loss increased after 21 (34.0 ± 0.3%) and 28 days (27 ± 6%), showing intermediate values between PLA/5Mg and PLA/5MgTT. After 28 days, the weight loss increased more rapidly compared to PLA/5MgTT and, at the end of the experiment, it reached values (91 ± 1%) comparable to those observed for PLA/Mg. The kinetic behavior of mass loss in the composite is different than in PLA, even if all materials are visibly disintegrated after 49 days of immersion.

PLA/5MgPEI with physical encapsulation and PLA/5MgTT with Mg thermal treatment were found to be the most resistant to degradation. This behavior may be attributed to the enhanced resistance conferred to the polymer by the PEI treatment of the magnesium particles. Ferrandez-Montero et al. [2] demonstrated the significant protective capacity of PEI; specifically, magnesium particles modified through PEI adsorption remained stable and protected against corrosion. Thus, it can be assumed that the interfacial adhesion between the polymer matrix and the Mg microparticles modulated by PEI presence effectively inhibits magnesium degradation to a greater extent. The treated magnesium incorporated into the polymers may have initially limited the mass loss during the first few weeks, thereby increasing the resistance of the polymer films to degradation. Similar results were reported by Xu et al. [33], who observed a more rapid decrease in the thickness of PLA fibers after prolonged exposure to alkaline conditions.

The thermally treated magnesium (MgTT) incorporated into the polymer decreased the mass loss, and this effect can be due to the formation of crystalline MgO at the selected treatment temperature.

#### 3.2.3. Water Absorption

**Figure 3:**
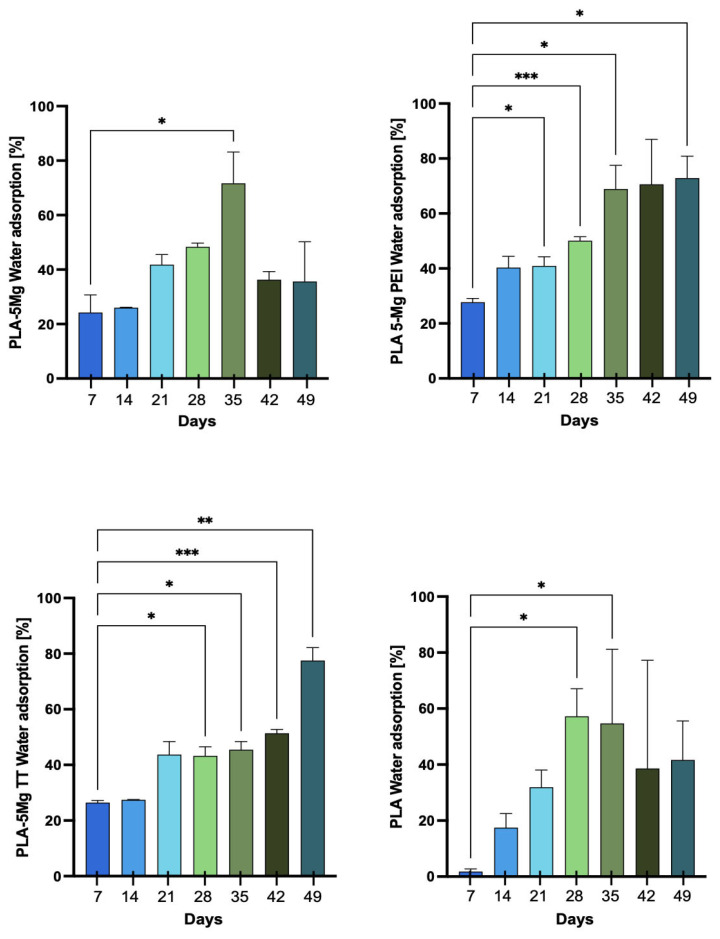
Water absorption percentage for PLA, PLA/5Mg, PLA/5MgTT, and PLA/5MgPEI PLA films as functions of degradation time in PBS at 60 °C. Results are expressed as mean ± SD of three independent experiments. The significance of the differences is indicated as follows: * = *p* < 0.05; ** = *p* < 0.01; *** = *p* < 0.001.

Figure 3 shows the water adsorption measurements of the samples, expressed as the mean percentage from the different specimens. Pristine PLA exhibited low water adsorption up to 14 days, followed by a marked increase after 14 days and a further rise after 28 days, reaching 77 ± 1% at 42 days. This represents the maximum value recorded throughout the degradation process. While pristine PLA was not significantly affected by degradation, water adsorption remained limited; however, once the material began to flake, it started to adsorb a greater amount of water. The high adsorption values observed at 42 days can be attributed to the almost complete degradation of the polymer, which had developed a mushy consistency, favoring further water uptake. All composites containing magnesium particles exhibited higher percentages of water adsorption due to the hydrophilic nature of magnesium and increased measured values of water contact angles in the composites with respect to pristine PLA [19]. PLA/5Mg, in particular, showed an early increase in water adsorption after just 7 days (24 ± 6%), continuing to rise until 35 days (72 ± 11%). Similar values were obtained for composites with surface-treated magnesium particles.

#### 3.2.4. Thermal Degradation Properties

**Figure 4:**
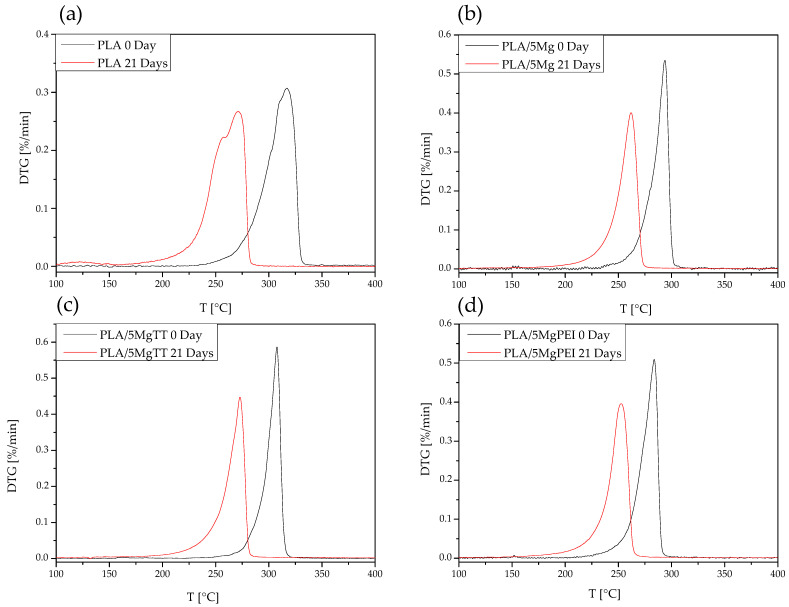
DTG curves of pristine and degraded PLA (**a**), PLA/5Mg (**b**), PLA/5MgTT (**c**), and PLA/5MgPEI PLA films (**d**) after 21 days of degradation time in PBS at 60 °C.

Taking into account the mass loss measurements, TGA was analyzed after 21 days of degradation. Figure 4 shows the DTG curves of pristine PLA, PLA/5Mg, PLA/5MgTT, and PLA/5MgPEI films compared with the degraded samples after 21 days of immersion in PBS at 60 °C. The presence of Mg particles within the PLA polymer matrix in as-prepared films reduced the maximum thermal degradation temperature from 317 °C for pristine PLA to 308 °C for PLA/5MgTT, 294 °C for PLA5/Mg, and 284 °C for PLA5/MgPEI [19].

After 21 days of degradation, a shift in the thermograms towards lower temperatures is observed: all samples exhibited decreased thermal degradation peaks. In particular, PLA decreased at 284 °C, with a two-step decomposition process and a lower starting degradation step: 282 °C for composites with MgTT, 275 °C for composites with Mg, and 267 °C for composites with MgPEI. This is an indication that the PLA polymer chains have been degraded. Neat PLA films also exhibited a secondary peak at lower temperatures, reaching a maximum at approximately 259 °C. This peak is presumably associated with the degradation of PLA oligomers formed at the outermost surface of the specimens [34].

#### 3.2.5. Raman Spectroscopy

**Figure 5:**
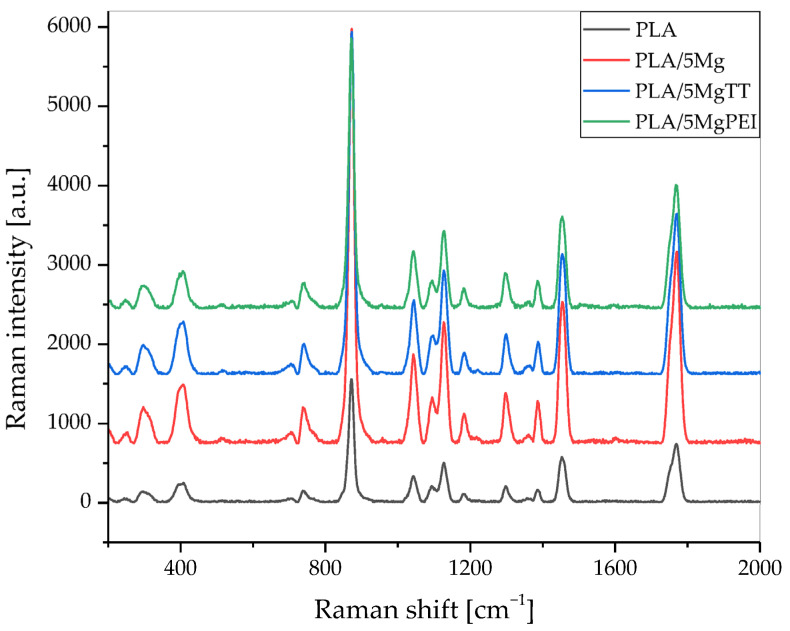
Raman spectra of PLA, PLA/5Mg, PLA/5MgTT, and PLA/5MgPEI films recorded in the spectral region of 200–2000 cm^−1^ (as-prepared).

**Figure 6:**
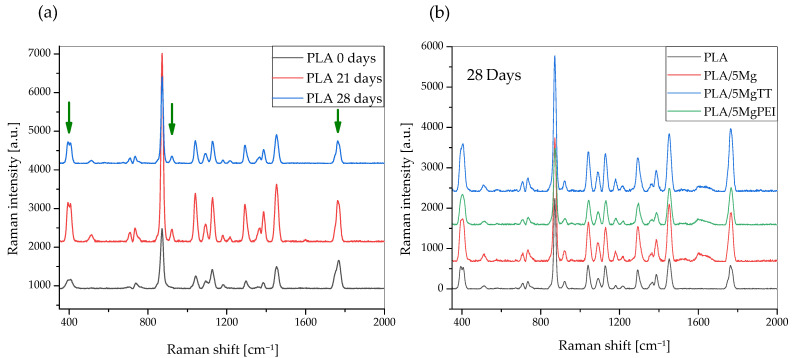
Raman spectra of the pristine PLA polymer films and hydrolytically degraded samples (**a**) and PLA and composites after 28 days of degradation time, in the spectral region of 350 to 2000 cm^−1^ (**b**).

Raman spectra of the as-prepared materials are shown in Figure 5, while Figure 6 compares the Raman spectra of as-prepared samples with the degraded films in the spectral region of 350 to 2000 cm^−1^. In the obtained spectra, several peaks are attributed to PLA polymer film [35,36,37], while the other peaks can be associated with the polymer degradation resulting from PBS immersion under the selected temperature and pH conditions. Figure 5 shows that the presence of magnesium particles does not alter the Raman spectra of PLA, as polymer and composites exhibit the same signals within the analyzed region.

Table 1 shows the wavenumber ranges associated with the functional groups present and characteristics of PLA [35,36,37,38]. Our results also confirm that the melting process and parameters used during the processing do not induce specific modifications in the main Raman peaks of PLA.

Raman spectroscopy is a powerful and important technique that permits one to investigate the structure of PL-based materials. However, Raman studies of PLA composites are rare. A detailed analysis of the previously published literature demonstrated that the Raman spectra of PLA-based materials strongly depend on polymer crystallinity degree [34] and, in general, on structural changes that can be modified during accelerated hydrolytic degradation experiments. Raman spectra showed the most intense line at ca. 870 cm^−1^, which was assigned to νC-COO stretching [36,37]. A second Raman line of low intensity was observed in the range of 920–908 cm^−1^ in the spectra of degraded films (green arrow) and was absent in all pristine films, as shown in Figure 6 [39]. Due to flexural C–H bond vibration, representative of the crystalline structure of PLA [39], it increases with the degradation time for pristine PLA polymer film and Mg-based materials [40]. Figure 6a shows a comparison of the Raman spectra of the pristine PLA polymer films with hydrolytically degraded materials, while Figure 6b shows Raman spectra of hydrolytically degraded PLA and composite films after 28 days of degradation time, in the spectral region of 350 to 2000 cm^−1^. Raman spectra of the pristine PLA polymer films, hydrolytically degraded samples, PLA, and composites after 28 days of degradation time, in the spectral region of 350 to 2000 cm^−1^, are shown. The band at 400 cm^−1^ (green arrow) appeared broad in the pristine PLA film, as in all amorphous PLA films, while during the degradation the band is resolved and split into two peaks, with the intensity increasing during the degradation time with increasing crystallinity. Mg-based composites showed a similar trend. An important difference can also be observed in the C=O carbonyl band at 1750 cm^−1^ (green arrow), which can be split into three main peaks, and during the degradation the intensity changed. We evaluated the variation in the relative intensity of the Raman band centered at approximately 405 cm^−1^ to investigate changes in the crystallinity of PLA polymer films induced by hydrolytic degradation, as this intensity ratio is strongly correlated with crystallinity, as previously suggested by Liubimovskii et al. [38]. The intensity ratio I_405_/I_874_ monotonously increases with the hydrolytic degradation time in all samples, as previously described [38], from 0.31 for PLA until 0.48 after 28 days of immersion. This variation can be due to both the erosion of the amorphous parts and a crystallization of the amorphous phase, taking into account the characteristics of the PLA polymer, which is almost completely amorphous at the beginning of the test. The degradation mechanism can be explained firstly with a random hydrolytic scission of ester bonds that proceeds with the diffusion of water into the amorphous regions. As the degradation proceeds, the degree of crystallinity tends to increase.

### 3.3. PLA/Mg Composite Films Are Suitable for hBM-MSCs Culture

**Figure 7:**
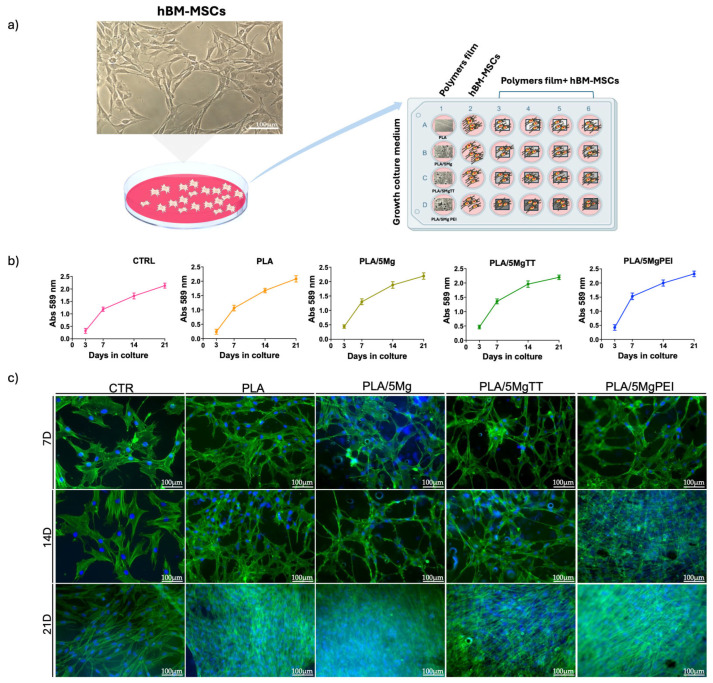
hBM-MSCs on CTR, PLA, PLA/5Mg, PLA/5MgTT, PLA/5MgPEI: viability and morphology. (**a**) Representation of study plan: left—representative brightfield image of hBM-MSCs on TCP, scale bar = 100 μm; right—culture of hBM-MSCs on neat PLA and on PLA-composite films for 21 days; CTR: stem cells on TCP/glass coverslip in the growth culture. Polymer films without stem cells were evaluated. (**b**) MTT assay of hBM-MSCs on PLA, PLA/composite films and on TCP at 3, 7, 14, and 21 days. Data are representative of three independent experiments that yielded similar results. (**c**) Representative images of immunostaining: nuclei (4′,6-diamidino-2-phenylindole, DAPI, blue) and F-Actin (Alexa-fluor-488 Phalloidin, green) at 7, 14, and 21 days. Images were acquired with fluorescence microscopy (Eclipse-TE2000-S, Nikon), using the F-View II FireWireTM camera (Soft Imaging System, Olympus, Germany, version 2.5, Accessed in 2006). Scale bar = 100 μm.

We evaluated the biocompatibility of PLA, PLA/5Mg, PLA/5MgTT, and PLA/5MgPEI films for the long-term culture of hBM-MSCs by seeding cells on polymer films in growth medium. As experimental control (CTR), hBM-MSCs were seeded on TCP/glass coverslips (Figure 7a). Namely, after 3, 7, 14, and 21 days of culture, cell viability was analyzed by MTT assay, and cell morphology by immunofluorescence. The MTT assay showed a similar mitochondrial dehydrogenase activity for hBM-MSCs on PLA, PLA/5Mg, PLA/5MgTT, and PLA/5MgPEI films and CTR cells over the time of culture (Figure 7b). No toxicity signs were observed by microscopy visual inspection. These results demonstrated the biocompatibility of these biomaterials to hBM-MSCs. Next, we analyzed the effect of PLA, PLA/5Mg, PLA/5MgTT, and PLA/5MgPEI films on the morphology of hBM-MSCs, by evaluating the cytoskeleton microfilament of F-actin organization. In all polymer films, the cell F-actin fibers, depicted in green in Figure 7c, were organized as straight, cable-like structures that traversed the cytoplasm, with a preferential orientation along the main longitudinal axis of the cell, similar to the arrangement seen in CTR cells. However, compared to the typical fibroblast-like morphology, hBM-MSCs cultured on PLA-derived films exhibited an elongated shape and more complex cell–cell interactions (Figure 7c). Notably, the cell morphology began to differ after day 7 of culture and was maintained throughout the culture period (Figure 7c).

### 3.4. Osteogenic Differentiation of PLA/Mg-Dervative Films on hBM-MSCs

**Figure 8:**
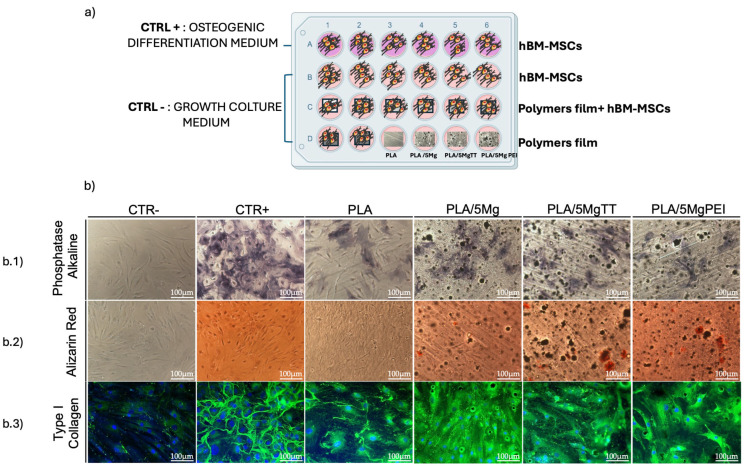
The osteogenic differentiation of hBM-MSCs on PLA, PLA/5Mg, PLA/5MgTT, and PLA/5MgPEI films. (**a**) Schematic of the plan: CTR+, stem cells grown with osteogenic differentiation medium; CTR−, stem cells cultured on TCP/GC in growth culture medium; hBM-MSCs cultured on PLA and PLA-composite films in growth culture medium. The films’ interference without stem cells was also evaluated. All cultures were performed for 21 days. (**b.1**) Representative brightfield images of alkaline phosphatase staining of CTR−, CTR+, and hBM-MSCs cultured on PLA and PLA/5Mg-based films in the growth culture medium. (**b.2**) Brightfield representative images of Alizarin Red staining of CTR−, CTR+, and hBM-MSCs cultured on PLA and PLA/5Mg-based. Scale bar = 100 μm. (**b.3**) Fluorescence representative images of type I collagen (green) and nuclei (DAPI, blue) of CTR−, CTR+, and hBM-MSCs cultured on PLA and PLA/5Mg-based films in the growth culture medium.

Given the osteoinductive potential of Mg in osteogenesis, we investigated the effect of PLA, PLA/5Mg, PLA/5Mg TT, and PLA/5Mg PEI polymer films on the osteogenic differentiation in hBM-MSCs cultured in growth medium, without the addition of soluble osteogenic inducers. Control experiments were carried out by culturing hBM-MSCs on TCP or coverslips with growth medium (CTR-) and osteogenic medium (CTR+). After 21 days, the duration required for in vitro osteogenic differentiation, we analyzed the expression of osteogenic markers: calcium deposition via Alizarin Red S (ARS) staining, alkaline phosphatase (ALP) activity using BCIP/NBT staining, and type I collagen expression by immunofluorescence (Figure 8b).

The expression of ALP is critical for the function of osteoblasts, the specialized cells responsible for bone formation. ALP plays a pivotal role in the mineralization process of bone by hydrolyzing pyrophosphate, a known inhibitor of mineral deposition [41]. In our system, ALP staining was strongly positive in hBM-MSCs cultured with a specific osteogenic differentiation medium, while it was undetectable in multipotent human mesenchymal/stromal cells cultured in standard conditions on TCP and only slightly detectable on PLA film (Figure 8(b.1)). Conversely, we observed an increase in ALP expression in hBM-MSCs seeded on PLA/5Mg, PLA/5MgTT, and PLA/5MgPEI, with the most intense signal observed in PLA/5MgTT films (Figure 8(b.1)). No ALP staining was detected in films in the osteogenic medium without stem cells.

These results were supported by the measures of calcium deposits through the incubation of the cells with Alizarin Red, the specific histological dye commonly used to detect calcium deposits in differentiated osteocytes. We observed positive staining in hBM-MSCs seeded on PLA/Mg-derived polymer films and in cells cultured with osteogenic differentiation medium (Figure 8(b.2)). An Alizarin Red signal was slightly detectable in hBM-MSCs seeded on PLA film (Figure 8(b.2)). In contrast, there was no staining in films in the osteogenic medium without stem cells and in stem cells cultured on TCP with the standard medium. A further validation of these results was obtained by the immunostaining of the osteogenic marker type I collagen, which is a structural component of the organic bone matrix, primarily synthesized by osteoblasts. In Figure 8(b.3), we found a good expression of type I collagen in hBM-MSCs seeded on PLA/5Mg, PLA/5MgTT, and PLA/5MgPEI, as well as in hBM-MSCs cultured on TCP in the presence of osteogenic differentiation medium, whereas little expression was observed in cells on PLA film (Figure 8(b.3)). However, no staining was detected in films in the osteogenic medium without stem cells and in stem cells cultured on TCP with the standard medium. Collectively, these results highlight the osteogenic potential of PLA/5Mg, PLA/5MgTT, and PLA/5MgPEI in promoting osteogenic differentiation in hBM-MSCs.

## 4. Conclusions

In this study, we investigated the effect of magnesium incorporation and surface modification on the accelerated hydrolytic degradation of PLA. All samples underwent complete degradation after 49 days of immersion at 60 °C, with all the samples in the rubbery state, although their degradation kinetics differed, with the PLA/5MgTT composite showing a slower degradation rate.

We confirm that Raman spectroscopy is an effective tool for monitoring structural changes in PLA-based materials. Specifically, we propose using the ratio of peak intensities at approximately 405 cm^−1^ and 870 cm^−1^ as an indicator of crystallinity in both neat PLA and Mg-based composites. Accelerated hydrolytic degradation leads to an increase in crystallinity, accompanied by corresponding changes in the thermal behavior of the samples, including a shift in thermal degradation temperature.

Collectively, these findings highlight the osteogenic potential of PLA/Mg-derived films in promoting the osteogenic differentiation of stem cells. Our results not only confirm previous findings (Argentati et al., 2022) [19] showing the pro-osteogenic effect of PLA/5Mg-based films on human adipose-derived stem cells, but also demonstrate a comparable osteogenic response for PLA/5Mg, PLA/5MgTT, and PLA/5MgPEI when tested on human bone marrow-derived mesenchymal stromal cells (hBM-MSCs). This contrasts with prior data suggesting a dominant effect of PLA/5MgTT.

Overall, our results underscore the importance of selecting the appropriate stem cell type when evaluating the osteoinductive potential of biomaterials.

## Figures and Tables

**Table 1:** Wavenumber ranges (${cm}^{-1}$) and general functional groups for each corresponding range of PLA films.

$\mathrm{Wavenumber}\;\mathrm{Range}\;({cm}^{-1})$	Functional Groups
291–309	δCOC
398–411	δCOO
650–677–711	δC=O; γC=O (weak C=O groups)
736–760	δC=O; γC=O (modearate C=O groups)
870	νC–COO
908–920	νC–C
1045	C–CH_3_
1090	C–O–C
1270–1300	CH + COC
1300–1360	C–CH_3_
1452	CH_3_
1749–1773	C=O (strong C=O groups)

## Data Availability

The original contributions presented in this study are included in the article. Further inquiries can be directed to the corresponding author.
